# Phylogeographical Analysis Reveals the Historic Origin, Emergence, and Evolutionary Dynamics of Methicillin-Resistant *Staphylococcus aureus* ST228

**DOI:** 10.3389/fmicb.2020.02063

**Published:** 2020-08-26

**Authors:** Mohamed M. H. Abdelbary, Edward J. Feil, Laurence Senn, Christiane Petignat, Guy Prod’hom, Jacques Schrenzel, Patrice François, Guido Werner, Franziska Layer, Birgit Strommenger, Annalisa Pantosti, Monica Monaco, Olivier Denis, Ariane Deplano, Hajo Grundmann, Dominique S. Blanc

**Affiliations:** ^1^Service of Hospital Preventive Medicine, Lausanne University Hospital and University of Lausanne, Lausanne, Switzerland; ^2^Division of Oral Microbiology and Immunology, Department of Operative Dentistry, Periodontology and Preventive Dentistry, RWTH Aachen University Hospital, Aachen, Germany; ^3^The Milner Centre for Evolution, Department of Biology and Biochemistry, University of Bath, Bath, United Kingdom; ^4^Institute of Microbiology, Lausanne University Hospital and University of Lausanne, Lausanne, Switzerland; ^5^Bacteriology Laboratory, Division of Infectious Diseases, Geneva University Hospitals, Geneva, Switzerland; ^6^National Reference Centre for Staphylococci and Enterococci, Division of Nosocomial Pathogens and Antibiotic Resistances, Department of Infectious Diseases, Robert Koch Institute, Wernigerode, Germany; ^7^Department of Infectious, Parasitic and Immune-Mediated Diseases, Istituto Superiore di Sanità, Rome, Italy; ^8^National Reference Centre-Staphylococcus aureus, Department of Microbiology, Hôpital Erasme, Université libre de Bruxelles, Brussels, Belgium; ^9^Laboratory of Microbiology, CHU UCL Namur, Université catholique de Louvain, Yvoir, Belgium; ^10^Department of Infectious Diseases Epidemiology, The University of Groningen, Groningen, Netherlands

**Keywords:** MRSA, ST228, WGS, phylogeography, phylogeny, genomic epidemiology, epidemiology

## Abstract

**Background:**

Methicillin-resistant *Staphylococcus aureus* (MRSA) is a common healthcare-associated pathogen that remains a major public health concern. Sequence type 228 (ST228) was first described in Germany and spread to become a successful MRSA clone in several European countries. In 2000, ST228 emerged in Lausanne and has subsequently caused several large outbreaks. Here, we describe the evolutionary history of this clone and identify the genetic changes underlying its expansion in Switzerland.

**Materials and Methods:**

We aimed to understand the phylogeographic and demographic dynamics of MRSA ST228/ST111 by sequencing 530 representative isolates of this clone that were collected from 14 European countries between 1997 and 2012.

**Results:**

The phylogenetic analysis revealed distinct lineages of ST228 isolates associated with specific geographic origins. In contrast, isolates of ST111, which is a single locus variant of ST228 sharing the same *spa* type t041, formed a monophyletic cluster associated with multiple countries. The evidence points to a German origin of the sampled population, with the basal German lineage being characterized by *spa* type t001. The highly successful Swiss ST228 lineage diverged from this progenitor clone through the loss of the aminoglycoside-streptothricin resistance gene cluster and the gain of mupirocin resistance. This lineage was introduced first in Geneva and was subsequently introduced into Lausanne.

**Conclusion:**

Our results reveal the radiation of distinct lineages of MRSA ST228 from a German progenitor, as the clone spread into different European countries. In Switzerland, ST228 was introduced first in Geneva and was subsequently introduced into Lausanne.

## Introduction

*Staphylococcus aureus* colonizes the anterior nares of about 30% of healthy humans. However, methicillin-resistant *Staphylococcus aureus* (MRSA) remains a major global cause of healthcare associated infections (Stefani et al., 2012; Aanensen et al., 2016). In addition, infections due to MRSA are associated with higher mortality rates than infections caused by methicillin-susceptible strains and result in increased lengths of hospital stays as well as associated health care costs (Barrasa-Villar et al., 2017; Hassoun et al., 2017). Successful reduction of MRSA infection rates depends on preventing MRSA transmission and detecting and containing outbreaks (Byrne and Wilcox, 2011). Understanding the settings and circumstances under which MRSA spread in the hospital setting and in the community is central to design new strategies to reduce transmission.

Molecular typing methods play an important role in understanding the epidemiology of MRSA and tracking different outbreaks (Urwin and Maiden, 2003). For example, the multilocus sequence typing (MLST) method have assigned most of MRSA isolates into a limited number of clonal lineages (Robinson and Enright, 2003). In addition, it was previously shown that certain MRSA clones have disseminated worldwide, while others were found in geographically limited regions (Monecke et al., 2011). However, the epidemiology of MRSA is dynamic, and clonal replacement of predominant clones within a given hospital has been widely reported (Kuhn et al., 2007; Albrecht et al., 2011; Campanile et al., 2015; Zarfel et al., 2016). In contrast to MLST, whole-genome sequencing (WGS) provides high discriminatory power that reveals the diversity among isolates of the same clone (Sequence Type). Such data are proving very powerful for epidemiological surveillance on continental scales down to a single hospital, and are informing more efficient infection control (Aanensen et al., 2016; Raven et al., 2019).

Methicillin-resistant *Staphylococcus aureus*-ST228 was first described in Germany in 1992, and was initially called the southern German or Italian clone (Witte et al., 1997; Witte, 1999; Monecke et al., 2011). This clone first emerged in the Lausanne university hospital in 2000 and caused a long-term outbreak that lasted between 2008 and 2012. Senn et al. recently used WGS to study this outbreak and argued that carriers of this clone may have gone unrecognized in the hospital due to an atypically high rate of enteric carriage, combined with high transmissibility (Vogel et al., 2012; Senn et al., 2016).

In this study, we significantly expand on this work, and place the Lausanne outbreak within a phylogeographic context, by generating WGS data for 302 additional isolates representing a wide temporal and geographic spread. We combined these data with those of Senn et al. (2016) to give a total dataset of 530 isolates. These data point to a German origin of this clone and reveal detailed geographic and temporal information on subsequent inter- and intra-national spread and significant genomic events occurring during this spread.

## Results

### Phylogenetic Reconstruction of the ST228

We analyzed genome sequences of 530 MRSA isolates that were collected from health-care institutions of 14 European countries between 1997 and 2012 (Supplementary Table S1). *In silico* MLST analysis revealed that 487 isolates were assigned to ST228, while 43 belonged to ST111 (a single locus variant of ST228 sharing the same t041 *spa* type) (Supplementary Table S1). Mapping the 530 MRSA genomes to the N315 reference genome (accession number NC_002745.2) and subsequent Gubbins analysis identified 16 diverse genes exhibiting between 4 and 17 SNPs (Supplementary Table S2). These included surface antigens such as *spa*, adhesins such as *clfA*, *clfB*, *ssrA*, and MBL fold metallo-hydrolase genes. It is likely that this diversity reflects a history of duplication/deletion or recombination at these loci.

**FIGURE 1:**
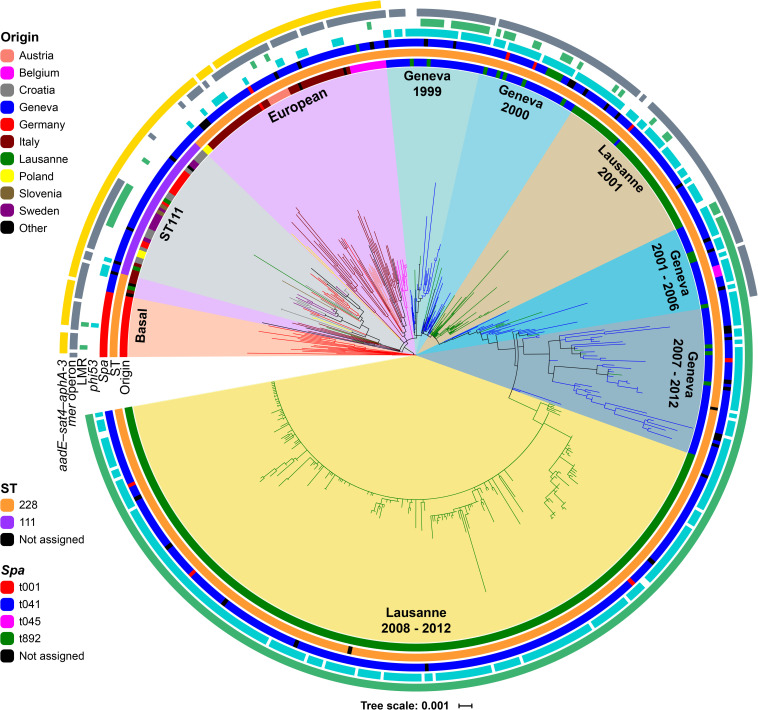
Rooted maximum likelihood (ML) phylogenetic tree of the 530 ST228 (*n* = 485) and ST111 (*n* = 43) MRSA isolates based on 3,899 core-genomes SNPs. The tree was rooted by using the distantly related *S. aureus* N315 as an outgroup. Branch colors represent country/cities of isolation. Rings from inner to outer; first ring (*Origin*) represent the geographical origin, second ring (*ST*) is assigned to the different STs, third ring (*spa*), fourth ring (*phi53*) detected, fifth ring (*LMR*) mutation responsible for the low level mupirocin resistance phenotype, sixth ring (*mer operon*) shows the isolates harboring the mercury operon gene cluster, and seventh ring (*aadE-sat4-aphA-3*) represents the acquisition of the gene cluster.

The phylogenetic analysis identified 3,899 SNPs within the core-genomes of the 530 isolates, after exclusion of mobile genetic elements (MGEs), recombinant and repetitive regions. These core-genome SNPs were used to reconstruct the phylogeny of all ST228 and ST111 isolates using a maximum-likelihood (ML) approach (Figure 1). Based on the phylogenetic tree, isolates were clustered into nine distinct clades that broadly correlated with geographical origin. The ST111 isolates formed a distinct cluster that was not obviously associated with a single geographic origin. The basal clade consisted exclusively of German ST228 isolates with *spa* type t001 (*n* = 18). Other clades of ST228 isolates also showed geographical association based on the country of origin. For example, the European clade included the Italian ST228 isolates (*n* = 41), which form two distinct sub-clades; 2 Polish isolates and 7 Austrian isolates are closely related to these Italian isolates. All Belgian ST228 isolates (*n* = 12) made up a distinct cluster among the European clade (Figure 1). Interestingly, the Swiss ST228 isolates (*n* = 402) corresponded to a monophyletic cluster that was divided into six clades (Geneva 1999, Geneva 2000, Lausanne 2001, Geneva 2001–2006, Geneva 2007–2012, and Lausanne 2008–2012) with a basal clade composed of Geneva isolates (Geneva 1999). Only three Swiss isolates were in other clusters. This suggests that the rate of transmission of MRSA ST228 into, and out of, Switzerland has been relatively low. Two Swiss isolates correspond to ST111 (La233 and Vd466) and clustered in clade ST111, and one isolate La005 clustered in the European clade close to an Italian isolate IT705. Of note, the epidemiological data revealed that isolate La005 was retrieved from a patient who was transferred from Italy. Clade Lausanne 2008–2012 (Figure 1) consists of isolates that were previously investigated and represent the Lausanne outbreak involving over 1600 patients from 2008 to 2012 (Senn et al., 2016). The phylogenetic structure of this clade showed 7 branches with low level of variation that was concordant with the previous observations, which suggested a clonal dissemination of this lineage within the university hospital of Lausanne (Senn et al., 2016). In contrast to ST228, the ST111 clade had a weak signal of geographical clustering, suggesting frequent transmission of ST111 strains between European countries.

### Genomic Diversity and Antibiotic Resistance

Mobile genetic elements are a substantial constituent of *S. aureus* genomes, and they may mediate the emergence of new MRSA lineages (Jamrozy et al., 2017). Pan-genome analysis detected a total of 3,271 genes among the 530 MRSA isolates, 2,531 (77%) of these were core genes (present in >95% of the isolates) while the remaining 740 genes (23%) are variably present or absent and hence correspond to the accessory genome (Supplementary Figure S1). Furthermore, 609 of the accessory genes were present in <15% of the isolates.

The number of putative prophages identified with each genome ranged from three to nine, only three of these were intact prophages and these were the most abundant. The presence of φ53 prophage (accession number NC_007049) was detected in 71% (*n* = 377) of isolates, which was mainly associated with the six Swiss ST228 clades (*n* = 356) (Figure 1), while φJB prophage (accession number KT344895) was linked to the non-Swiss isolates. A previous study showed that φ53 and φJB were genetically distinct and φJB prophage was able to transfer antibiotic resistance plasmids at higher frequencies compared with φ53 prophage during transduction experiments (Varga et al., 2016). φPT1028-like prophage (accession number NC_007045.1) was present in all the genomes so it was probably present in the common ancestor of the ST228/111 clone.

All the antimicrobial resistance and virulence genes detected using the approaches described in Methods are listed in Supplementary Table S3. Remarkably, all the Swiss isolates lacked the *ant(6)-Ia*, *aph(3)-III* and *sat4* genes (the aminoglycoside-streptothricin resistance gene cluster) that encodes resistance to kanamycin/amikacin and streptothricin, while they were present in all remaining European ST228 and ST111 isolates. This gene cluster was part of a transposon structure, Tn5405, found previously on the chromosomes of staphylococci and other enterococci (Derbise et al., 1996; Werner et al., 2001). However, the bifunctional aminoglycoside modifying enzyme gene [*aac(6′)-Ie-aph(2″)-Ia*] was detected in all isolates (Supplementary Table S3). This gene confers high-level resistance to a wide range of aminoglycosides including gentamicin but, excluding streptomycin (Livermore, 2000).

Forty percent of isolates harbor the plasmid pTW20_1 that was previously described in MRSA ST239 (Holden et al., 2010). The pTW20_1 sequence was assembled on two contigs, one contig included the *qacA* gene (confers resistance to detergents and disinfectants: quaternary ammonia compounds – a marker for hospital adaptation) (Costa et al., 2013) and cadmium resistance operon (*cadA* and *cadD*), while the second contig carried genes encoding the mercury resistance operon (*merA*, *merB*, *merR*, *merT*). A variant of pTW20_1 that lacked the *mer* operon contig was detected in 55% (*n* = 296) of the isolates, the majority of which originated from Switzerland (230 from Lausanne and 39 from Geneva) and correspond to the clades Geneva 2007–2012 and Lausanne 2008–2012. However, this pTW20_1 variant (that lacked the *mer* operon) was detected in 27 isolates that were located in the Basal, the ST111 and the European clades and were collected from Germany (*n* = 11), Italy (*n* = 7), Croatia (*n* = 6), Sweden (*n* = 2), and Poland (*n* = 1) (Figure 1). These 27 isolates were collected between 1997 and 2012 and the oldest isolate (DE802) was collected in Germany during 1997 (Supplementary Table S1). We detected the β-lactamase operon (*bla*) in all 530 MRSA genomes that composed of *blaZ*, *blaR1* and *blaI* genes, which was inserted between *qacA* gene and cadmium resistance operon. Furthermore, mapping the genomes against the plasmid pTW20_1 sequence (29,585 bp; accession number FN433597) confirmed that those genomes which contained this variant (lacking the *mer* operon) had only 74% of aligned bases to the plasmid sequence, while the other genomes that carried this operon had an average of 96% aligned bases. Hence, these findings suggest that ST228 acquired two plasmid variants on different occasions and these lineages were circulating in the Swiss hospitals. A similar observation was previously described for MRSA ST239, where pTW20_1 was detected among three phylogenetic clades (Tong et al., 2015). Two of these clades carried pTW20_1 with *mer* operon, while the third clade carried the pTW20_1 variant that lacked *mer* operon.

The point mutation (V588F) in the chromosomal isoleucyl-tRNA synthase gene (*ileS*) that encodes low-level mupirocin resistance (LMR) was detected in 65% (*n* = 344) of isolates. Among these isolates 95% (*n* = 326) were of Swiss origin that were collected between 2001 and 2012 and distributed over the six Swiss phylogenetic clades (Figure 1). The entire clades Geneva 2001–2006, Geneva 2007–2012, and Lausanne 2008–2012 were composed of isolates with LMR, with the exception of isolate Ge348 that was collected in Geneva during 2004 (Figure 1). Furthermore, the point mutation V588F was detected in 18 non-Swiss isolates that were collected between 1999 and 2011 (Supplementary Table S3). The *iles-2* gene encoding high-level mupirocin resistance was detected in 26 isolates that were all from Switzerland.

### Demography and Phylogeographic Spread of ST228

**FIGURE 2:**
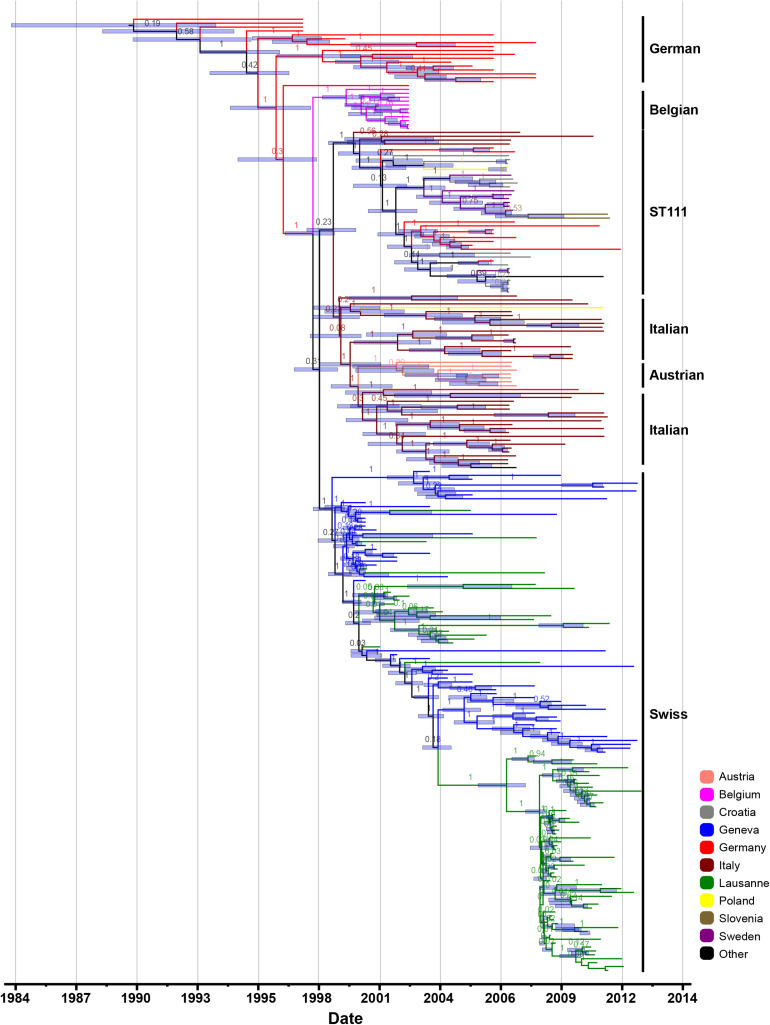
Maximum clade credibility (MCC) tree. Maximum clade credibility Bayesian phylogenetic tree reconstruction of the selected subset of 245 MRSA ST228 and ST111 MRSA genomes that were collected between 1993 and 2012. Tips of the tree are embarrassed by isolation dates; the time scale is shown at the base of the tree and countries/cities of origin are highlighted in different colors.

The root-to-tip regression analysis of all 530 genomes, and the sub-sample of 245 genomes (2,870 SNPs) both revealed a strong temporal signal (*R*^2^ = 0.68 and 0.58, respectively; *p* < 0.0001) (Supplementary Figure S2). Furthermore, we tested several demographic models on the sub-sample of 245 genomes and compared their marginal likelihood estimation (MLE). This revealed that the Hasegawa-Kishino-Yano (HKY) substitution model with relaxed uncorrelated molecular clock and a Bayesian skyline coalescent was the most supported combination (Supplementary Table S4). We estimated a mean mutation rate of 1.87 × 10^–6^ (95% highest posterior density interval (HPD), 1.63 × 10^–6^ to 2.13 × 10^–6^) substitutions per site per year, which is highly consistent with previous estimates for CC5 strains from the Western Hemisphere and other *S. aureus* clones (Glaser et al., 2016; Uhlemann et al., 2017; Challagundla et al., 2018). Based on this rate, the time of most common recent ancestor (TMRCA) of ST228 was estimated as 1988 (95% HPD, 1983 to 1992), and ST111 is estimated to have emerged in 1999 (95% HPD, 1998 to 2000). The TMRCA of the Swiss ST228 dates to 1998 (95% HPD, 1997 to 1999), and it was introduced first in Geneva and one month later it was circulated in Lausanne (Figure 2).

We then dated the acquisition and loss of various MGEs associated with antibiotic resistance. Our analysis suggests that the aminoglycoside-streptothricin resistance gene cluster was lost in 1989 (95% HPD, 1984 to 1993), while the acquisition of pTW20_1 plasmid variant (lacking the *mer* operon) and LMR were gained in 1990 (95% HPD, 1986 to 1993) and 1994 (95% HPD, 1991 to 1995), respectively.

**FIGURE 3:**
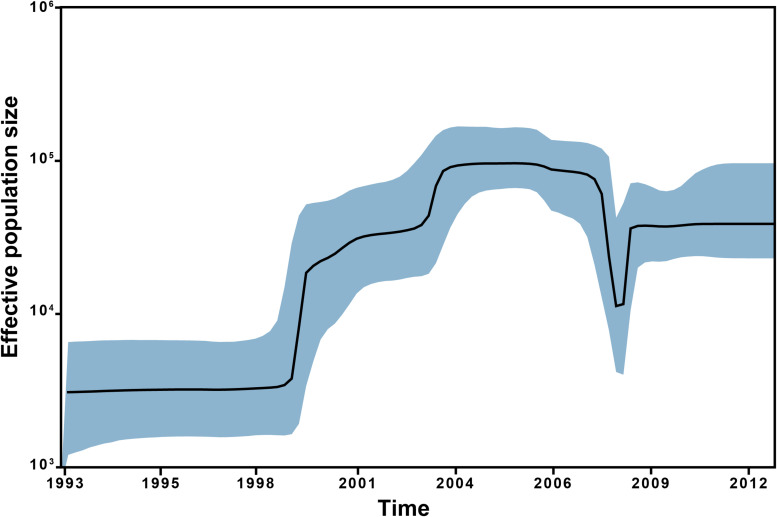
Bayesian Skyline plot of the analyzed selected subset of 245 MRSA ST228 and ST111 MRSA genomes. Bayesian Skyline plot with a relaxed molecular clock representing the effective population size of the ST228 over time with the shaded areas representing 95% confidence intervals.

The Bayesian skyline plot points to a steady effective population size of ST228 up until the first introduction into Switzerland in 1998 (Figure 3). Subsequently, a one order of magnitude increase in the ST228 population size was observed, which corresponds to the dissemination and expansion in Switzerland during the first outbreak in Geneva in 1999. The population size of ST228 continued to increase until a peak in around 2004, representing the different epidemic episodes in Geneva and Lausanne (Figure 3). During 2006, a sharp decline in ST228 population size was detected followed by a bottleneck in 2007. The introduction of ST228 isolates that lacked the *mer* operon into Switzerland gave rise to the last epidemic wave that lasted until 2012, when the ST228 population size became steady once again. These findings are concordant with our previous study (Senn et al., 2016), along with epidemiological data, which point to a decrease in MRSA ST228 incidence in Lausanne from 2005 till 2008, and the absence of large outbreaks in Switzerland after 2012. The inclusion of additional isolates from Geneva and Lausanne in the current study enabled a precise estimation of the demographic expansion of ST228 in Switzerland and revealed important details regarding the underlying genetic changes that are likely to have contributed to this spread.

**FIGURE 4:**
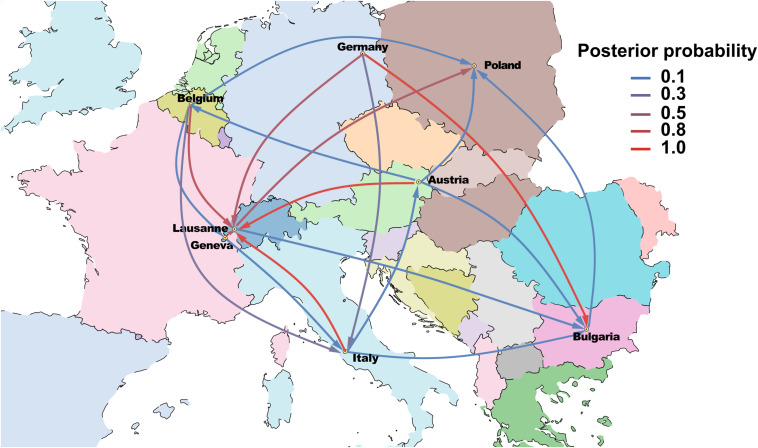
Phylogeographic reconstruction of ST228 representing the various transmission events among European countries. Lines are colored according to posterior probability support and arrows represent the general direction of the expansions.

The Bayesian phylogeographical analysis confirmed that Germany was the source of ST228, and that descendants of this progenitor populations were introduced into multiple countries. The spatial phylogenetic reconstruction (Figure 4) supports transmission from Germany to Bulgaria [Posterior probability (PP) = 1]. A direct introduction from Germany into Italy was observed but weakly supported (*PP* = 0.36) by the Bayesian stochastic search variable selection (BSSVS) analysis. Furthermore, we detected significant evidence of demographic expansion of ST228 from Geneva into Lausanne (*PP* = 0.97). In addition, transmission events from Austria, Belgium, Italy, and Germany into Lausanne were also statistically supported (*PP* = 0.98, 0.76, 0.99, and 0.67, respectively). These data implicate that ST228 has been introduced into Lausanne on at least five separate occasions.

## Discussion

In this study, we described the core-genome variation and the accessory gene profiles of 530 isolates of ST228/111 representing 14 European countries and 15 years of clonal spread. The WGS data provided detailed evidence concerning the epidemiological and evolutionary dynamics of this clone and dated key transmission events from the original progenitor population in Germany.

Our analyses show that recombination played a limited role in the evolutionary dynamics of ST228, and that the majority of the core genome variation detected arose instead through *de novo* mutation. These findings agree with previous study that estimated a low ratio of recombination to mutation events (0.0046) among strains of CC5, which included ST228 and ST111 (Challagundla et al., 2018). Exceptions included genes encoding surface proteins such as *spa*, *clfA* and *clfB*, which is consistent with the view that such genes are subject to diversifying selection pressure. High rates of homologous recombination have previously been observed in *S. aureus* adhesion genes (*clfA* and *clfB*) (Kuhn et al., 2006; Senn et al., 2016) and in surface antigens such as *spa* (Santos-Júnior et al., 2016).

Our pan-genome analysis revealed that several ST228 lineages have recently acquired or lost MGEs. Notably, all the Swiss isolates lacked the aminoglycoside-streptothricin resistance gene cluster [*ant(6)-Ia*, *aph(3)-III* and *sat4*]. The loss of this cluster may be explained by the fact that aminoglycosides are primarily used to treat infections caused by Gram-negatives and are rarely used for treating MRSA. The selective pressure to maintain the *ant(6)-Ia*, *aph(3)-III* and *sat4* gene cluster will be further reduced by the fact that an alternative gene cluster, *aac(6′)-Ie-aph(2″)-Ia*, which confers high-level resistance aminoglycosides (including gentamicin) was present in all of the isolates. We note that our Bayesian analysis revealed that the loss of the *ant(6)-Ia*, *aph(3)-III* and *sat4* gene cluster occurred in 1989 (95% HPD, 1984 to 1993).

Our data also reveal that the related clusters Geneva 2007–2012 and Lausanne 2008–2012 have lost the pTW20_1 plasmid, which harbors the *mer* operon (conferring resistance to mercury) and which is present in the majority of strains from various other origins (Figure 1). The strains in these clusters instead harbor a distinct variant of this plasmid which does not harbor the *mer* operon. However, this plasmid variant (lacking the *mer* operon) was detected in 27 non-Swiss isolates that were collected between 1997 and 2012 and located in the Basal, ST111 and European clades. Furthermore, our analysis revealed that the loss of this operon occurred in 1990 (95% HPD, 1986 to 1993), suggesting that the acquisition of this plasmid variant occurred prior to the introduction into Switzerland.

A third change in resistance profile evidenced from our data concerns the low-level mupirocin resistance (LMR), which is conferred by the V588F mutation in the *ileS* gene. The phylogenetic tree (Figure 1) shows that it arose on several occasions during the evolution of the ST228 clone; the oldest LMR isolate (DE805) being of German origin and collected during 1999. However, the great majority of isolates in the three monophyletic clades Geneva 2001–2006, Geneva 2007–2012 and Lausanne 2008–2012 showed this mutation. As argued by Senn et al. (2016) the presence of LMR probably resulted from the topical use of mupirocin for the decolonization of MRSA carriers in both hospitals and thus can help to explain this sustained outbreaks. It is interesting to note that both clades Geneva 2007–2012 and Lausanne 2008–2012 lacked the *mer* operon and showed the LMR mutation, suggesting that both mutations were at the origin of a successful lineage responsible for the two largest outbreaks in Geneva and Lausanne.

The mutation rate estimated here of 1.87 × 10^–6^ (95% HPD, 1.63 × 10^–6^ to 2.13 × 10^–6^) substitutions per site per year is highly consistent with previous estimates of the Swiss ST228 outbreak and with other MRSA STs (Harris et al., 2013; Hsu et al., 2015; Senn et al., 2016). In addition, a previous study that investigated the evolution of 598 CC5 genomes in the Western Hemisphere estimated three mutations rates for the entire CC5 [1.55 × 10^–6^ (95% HPD, 1.39 × 10^–6^ to 1.71 × 10^–6^), 1.52 × 10^–6^ (95% HPD, 1.36 × 10^–6^ to 1.68 × 10^–6^) and 1.55 × 10^–6^ (95% HPD, 1.39 × 10^–6^ to 1.70 × 10^–6^)], which were comparable to our estimation (Challagundla et al., 2018). The Bayesian skyline plot indicates that the expansion of the ST228 population was mainly due to its dissemination in Switzerland. In addition, the skyline analysis revealed a sharp decline of ST228 population size from 2006 to 2007, reflecting the spatiotemporal change of this clone within central Europe. This finding agrees with previous studies that reported a decreasing incidence of ST228. For example, a decreased in the incidence of ST228 in German hospitals was reported since 2001 (Werner et al., 2004; Albrecht et al., 2011; Layer et al., 2012, 2018). In Italy, the replacement of ST228 by ST22-IV clone was documented during 2000–2007 (Baldan et al., 2012).

Our Bayesian phylogeographic analysis traced the progenitor population of ST228 to Germany in 1988 (95% HPD, 1983 to 1992), and that the other lineages subsequently diverged from this population. This result is consistent with the original nomenclature of ST228 as southern German epidemic strain (Witte et al., 1997). Challagundla et al. shown that European strains of ST228 and ST111 represented early-branching of CC5 clade with *SCCmec* I and the TMRCA was estimated as the early 1970s [1973 (95% HPD, 1986 to 1993)]. However, the aforementioned study included only five ST228 and four ST111 strains and the estimated TMRCA represented strains of ST5 (*n* = 135), which might explain the divergence between our TMRCA estimate and that of by Challagundla et al. (2018). Our phylogeographic analysis provides strong support for some international transmission events, such as from Germany to Bulgaria (Figure 4), but only weak support for other routes such as Germany to Italy. This is likely due to the paucity of isolates from Italy recovered after 2005.

However, we find strong evidence that ST228 disseminated between Geneva and Lausanne from 1998 onwards. These data agree with a previous study showing that the ST228 was first introduced into Switzerland via Geneva in 1998 (De Angelis et al., 2011; Kelley et al., 2015). Here we also show strong evidence that ST228 has been introduced into Lausanne on four additional occasions, from Austria, Belgium, Italy, and Germany. These findings suggest that some ST228 sub-lineages that evolved from the ancestor lineage were possibly disseminating in Switzerland through patient transfer or traveling and migration.

A limitation of our study is the lack of ST228 isolates from the remaining three European countries (Spain, Slovenia, and Denmark) where ST228 had been previously detected (Robinson and Enright, 2003; Alm et al., 2014). However, it is unlikely that this has seriously confounded our analyses, as only a single outbreak was reported for each country. In addition, most isolates in our collection are from Geneva and Lausanne, which may have introduced sampling bias during the phylogeographic reconstruction. However, this bias was considered prior to the Bayesian analysis, and a subset of 245 genomes was used for estimating the evolutionary history of ST228. Hence, we are confident that our data and analysis is largely representative for the ST228/111 clone as a whole.

In contrast to ST228 isolates, isolates of ST111 from different countries clustered together suggesting single introduction followed by diversification in these countries, which is probably due to human travel within Europe. ST111 is highly prevalent only in Croatia and was occasionally found in other European countries such as Denmark, Italy, and Sweden (Budimir et al., 2010; Aschbacher et al., 2012). However, the detection of ST111 in these countries has been associated with single introduction through human travel (Bartels et al., 2007; Aanensen et al., 2016).

In summary, this study revealed that MGEs have played an important role in the evolution of ST228 MRSA. Our results suggest that Germany was the main reservoir for ST228 lineages, and the dissemination of descendant lineages occurred into different European countries shortly after they emerged in Germany. Phylogeographical reconstruction suggests multiple introductions of ST228 into Switzerland that occurred on several occasions rather than a diverse local population. To our knowledge, this is the first study that represents the phylogeographic history of the ST228 MRSA clone.

## Materials and Methods

### Bacterial Isolates

We included a total of 530 MRSA isolates that were collected from health-care institutions of 14 European countries (Supplementary Table S1). The Swiss isolates were collected from the university hospital of Lausanne (*n* = 266), from long-term care institutions near Lausanne (*n* = 25) and from the university hospitals of Geneva (*n* = 114). Among this collection, 228 isolates from Lausanne were included in a previous study (Senn et al., 2016). All isolates were healthcare-associated MRSA. Other European isolates originated from two studies of the European Staphylococcal Reference Laboratory Working Group (Grundmann et al., 2010, 2014).

### Whole Genome Sequencing and Phylogenetic Analysis

Whole-genome sequencing (WGS) was performed as previously described (Senn et al., 2016). Briefly, bacterial genomic DNA was extracted and sequenced using the Illumina HiSeq 2000 platform (San Diego, CA, United States) generating 100 base paired-end reads. *In silico* multi-locus sequence typing (MLST) analysis was performed via SRST2 pipeline using the sequence reads (Inouye et al., 2014). For the phylogenetic analysis, sequence reads were mapped against the distantly related *S. aureus* N315 reference genome (accession number NC_002745.2) using Stampy version 1.0 (Kuroda et al., 2001; Lunter and Goodson, 2011). The *S. aureus* N315 was chosen as reference due to being the closest sequenced genome to ST228 (both belong to CC5), to determine the ancestral node of ST228, and to ensure the reproducibility and comparability to the previously published study by Senn et al. (2016). Single nucleotide polymorphisms (SNPs) were called across the core-genome using SAMtools mpileup as previously described by Li et al. (2009) and Senn et al. (2016). The core-genome alignment was defined as nucleotide sites shared by all consensus sequences. Therefore, unmapped reads and sequences that were not present in all 530 genomes (including insertions/deletions and ambiguous sites) were not counted as part of the core-genome and were not included in the phylogenetic analysis. Similarly, SNPs falling within repetitive genomic regions and MGEs of N315 were excluded from the core-genome alignment (Supplementary Table S5). Briefly, the MGEs of N315 listed in Supplementary Table S5 were previously published by Kuroda et al. (2001) and their coordinates were identified using the annotation file, while the positions of repetitive regions were identified using the repeat-match algorithm, which is implemented in MUMmer package version 3.23 (Kurtz et al., 2004). Gubbins version 2.3.1 (Croucher et al., 2015) with default settings was used to identify regions of high SNPs densities and suspected recombination events within the investigated genomes. The final alignment of non-recombinant core-genome SNPs was generated and used to construct a maximum likelihood (ML) phylogenetic tree using PhyML that is implemented in Seaview software version 4.7 (Gouy et al., 2010). The ML tree was rooted by using the distantly related *S. aureus* N315 as an outgroup. Visualization and annotation of the tree was performed using iTOL^1^ (Letunic and Bork, 2011).

### Demographic and Phylogeographic Analyses

To investigate the temporal signal in the data set, analyses of the correlation between root-to-tip genetic distance and year of sampling were performed on the maximum-likelihood tree using TempEst version 1.5.3 (Rambaut et al., 2016). In order to reduce the confounding effects of geographical biases, and to make the analyses more computationally efficient, we subsampled 245 representative genomes of ST111 and ST228 based on their isolation date and country of origin (Supplementary Table S1). Using this subsample, we performed a Bayesian analysis of evolutionary rates, divergence times estimation and phylogeographic inference using BEAST version 1.10.4 (Suchard et al., 2018) in combination with BEAGLE library version 3.1.2 (Ayres et al., 2012). A concatenated core SNPs alignment of all 245 MRSA genomes was used to infer the TMRCA and to reconstruct the phylogeographic spread. BEAST analysis was performed using the general-time reversible (GTR) and the HKY substitution models with gamma distributed among-site rate variation with four rate categories. A strict and uncorrelated relaxed molecular clock model with constant size, exponentially growing and Bayesian skyline coalescent was applied. For an accurate estimation of population size and branch lengths, unambiguous constant (invariable) sites were also included in the xml files. Three BEAST runs were performed, and for each run 100 million steps were generated, and the chain sampled every 10,000 states. A marginal likelihood estimation (MLE) using path sampling and stepping stone sampling was calculated for each run and the average values were used to compare the different combinations of clock and tree models and choosing the best supported model parameters. LogCombiner version 1.10.4 was used to combine the trees and log files from the multiple runs with 10% burn-in. Tracer version 1.7.1 (Rambaut et al., 2018) was used to visualize the results, examine the effective samples size (ESS) and to reconstruct the Bayesian Skyline plot. A maximum clade credibility (MCC) tree from the combined trees was obtained using TreeAnnotator version 1.10.4. Discrete trait model was applied to study the phylogeographic spread and the dissemination of antibiotic resistance and virulence determinants across the phylogeny. The phylogeographic tree was constructed using the ST228 sequences and their geographic coordinates in latitude and longitude as discrete traits under an asymmetric diffusion model implemented in BEAST. SPREAD3 version 0.9.7 (Bielejec et al., 2016) was used to visualize the spatial phylogenetic reconstruction of ST228, and to calculating Bayes factors (BFs) and posterior probabilities (PPs) from BSSVS analysis.

### *In silico* Detection of Genetic Markers

Sequence reads were assembled *de novo* into contigs, using Velvet version 1.0.14 and VelvetOptimiser version 2.2.5 (Zerbino and Birney, 2008). The generated contigs were ordered using MAUVE version 2.4.0 (Darling et al., 2010) and then, annotated using Prokka pipeline version 1.13.0 (Seemann, 2014). These *de novo* assembled, and annotated genomes were used to analyze the Pan-genome via Roary version 3.11.2 (Page et al., 2015), while Phandango version 1.3.0 (Hadfield et al., 2018) was used to visualize the results. We determined the presence or absence of acquired resistance and putative virulence genes via ABRicate version 0.8.13^2^ pipeline with default settings. The public databases ARG-ANNOT (Gupta et al., 2014) and CARD (Jia et al., 2017) were used as references for detecting the antimicrobial resistance determinants, while the VFDB (Chen et al., 2005) and PlasmidFinder (Carattoli et al., 2014) databases were used for identifying the virulence factor genes and plasmid sequences, respectively. Putative prophage regions among the assembled genomes were predicted using PHASTER (Arndt et al., 2016).

## Data Availability Statement

All sequencing raw data have been deposited in the European Nucleotide Archive (ENA) under study number ERP117831.

## Author Contributions

MA carried out the entire analysis and wrote the manuscript. DB and EF were the initiators of the study and edited the manuscript. The remaining authors contributed to the isolates collection with demographic and epidemiological data and commented on the manuscript. All authors contributed to the article and approved the submitted version.

## Conflict of Interest

The authors declare that the research was conducted in the absence of any commercial or financial relationships that could be construed as a potential conflict of interest.

## Footnotes

1 http://itol.embl.de

2 https://github.com/tseemann/abricate

## Supplementary Material

FIGURE S1 Pan-genome alignment of the investigated 530 ST228/ST111 MRSA genomes using Roary.

FIGURE S2 Root-to-tip regression compared to sampling date as estimated by TempEst. **(A)** Temporal signal in the entire dataset of 530 ST228/ST111 MRSA genomes. **(B)** Temporal signal in the entire sub-sampled dataset of 245 ST228/ST111 MRSA genomes.

TABLE S1 Metadata of the 530 MRSA isolates included in this study.

TABLE S2 Gubbins results representing detected genes with a high number of SNPs.

TABLE S3 Resistance and virulence factors detected in the 530 MRSA genomes.

TABLE S4 Marginal likelihood estimation for the 245 MRSA genomes sub-sampled dataset under various coalescent priors using path sampling and stepping stone.

TABLE S5 Mobile genetic elements and repetitive regions identified in the reference genome N315.
