# Supplementary figures and images for: Phylogeographical Analysis Reveals the Historic Origin, Emergence, and Evolutionary Dynamics of Methicillin-Resistant Staphylococcus aureus ST228
Source: Front Microbiol. 2020 Aug 26;11:2063. doi: 10.3389/fmicb.2020.02063 (PMC7479193; doi:10.3389/fmicb.2020.02063)

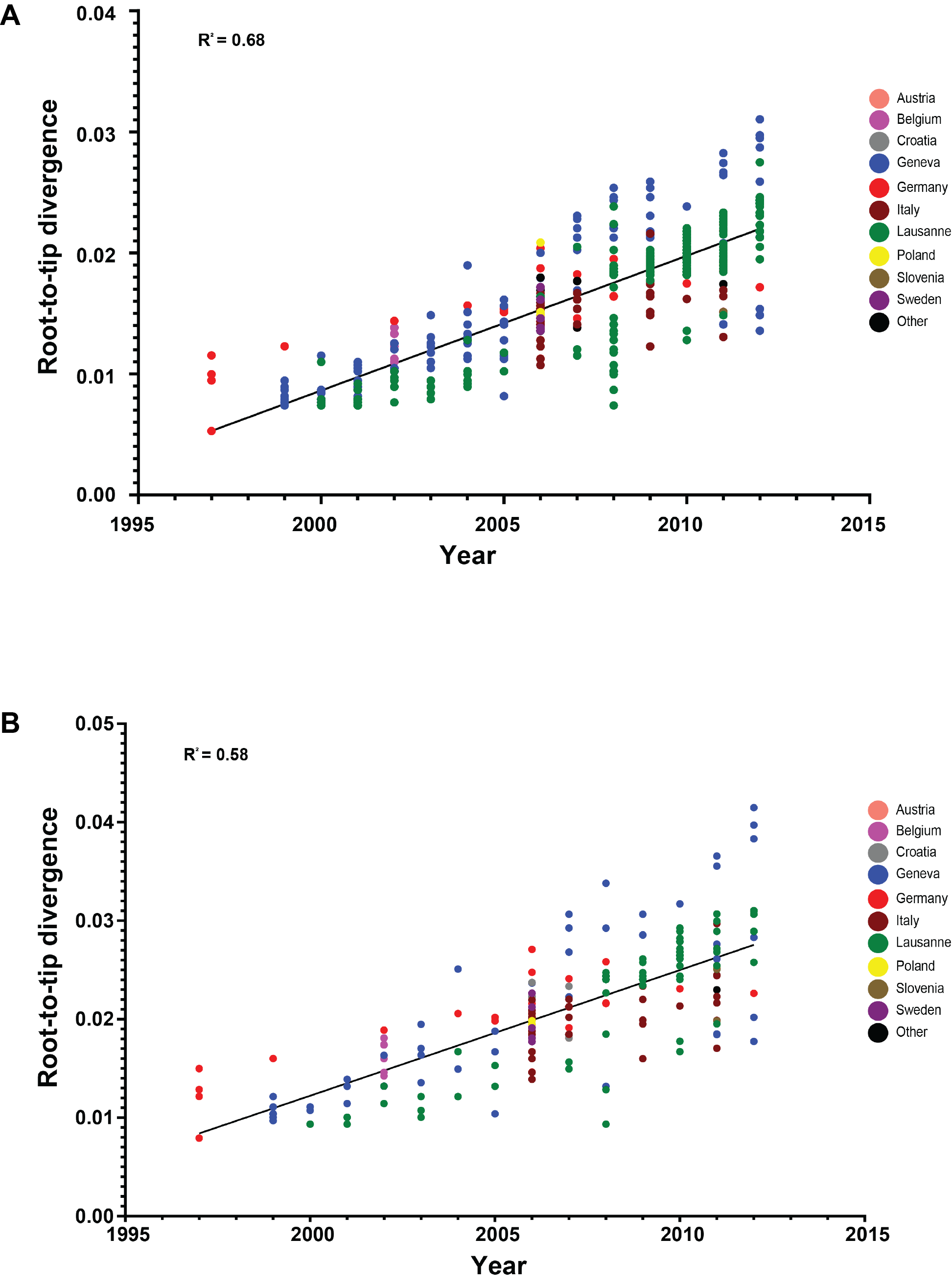

Supplement: FIGURE S2 — Root-to-tip regression compared to sampling date as estimated by TempEst. (A) Temporal signal in the entire dataset of 530 ST228/ST111 MRSA genomes. (B) Temporal signal in the entire sub-sampled dataset of 245 ST228/ST111 MRSA genomes. [file Image_2.TIF]
